# Single‐cell RNA sequencing in exploring the pathogenesis of diabetic retinopathy

**DOI:** 10.1002/ctm2.1751

**Published:** 2024-06-30

**Authors:** Xinzi Zhang, Fang Zhang, Xun Xu

**Affiliations:** ^1^ National Clinical Research Center for Eye Diseases Department of Ophthalmology Shanghai General Hospital Shanghai Jiao Tong University School of Medicine Shanghai China; ^2^ Eye Institute of Shanghai Jiao Tong University School Shanghai China; ^3^ Shanghai Key Laboratory of Ocular Fundus Diseases Shanghai China; ^4^ Shanghai Engineering Center for Precise Diagnosis and Treatment of Eye Diseases Shanghai China

**Keywords:** diabetic retinopathy, pathogenesis, single‐cell RNA sequencing

## Abstract

Diabetic retinopathy (DR) is a leading cause of irreversible blindness in the working‐age populations. Despite decades of research on the pathogenesis of DR for clinical care, a comprehensive understanding of the condition is still lacking due to the intricate cellular diversity and molecular heterogeneity involved. Single‐cell RNA sequencing (scRNA‐seq) has made the high‐throughput molecular profiling of cells across modalities possible which has provided valuable insights into complex biological systems. In this review, we summarise the application of scRNA‐seq in investigating the pathogenesis of DR, focusing on four aspects. These include the identification of differentially expressed genes, characterisation of key cell subpopulations and reconstruction of developmental ‘trajectories’ to unveil their state transition, exploration of complex cell‒cell communication in DR and integration of scRNA‐seq with genome‐wide association studies to identify cell types that are most closely related to DR risk genetic loci. Finally, we discuss the future challenges and expectations associated with studying DR using scRNA‐seq. We anticipate that scRNA‐seq will facilitate the discovery of mechanisms and new treatment targets in the clinical care landscape for patients with DR.

**Key points:**

- Progress in scRNA‐seq for diabetic retinopathy (DR) research includes studies on DR patients, non‐human primates, and the prevalent mouse models.
- scRNA‐seq facilitates the identification of differentially expressed genes, pivotal cell subpopulations, and complex cell‐cell interactions in DR at single‐cell level.
- Future scRNA‐seq applications in DR should target specific patient subsets and integrate with single‐cell and spatial multi‐omics approaches.

## Graphical abstract

scRNA‐seq provides novel insights into the pathogenesis of diabetic retinopathy, including identification of differentially expressed genes, characterisation of key cell subpopulations, construction of cell state transition trajectories and exploration of intricate cell‒cell communication.

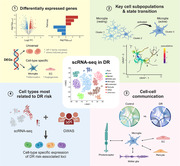

## 1 INTRODUCTION

Diabetic retinopathy (DR) is the major cause of vision impairment and blindness among the working‐age population.^1^ As one of the common complications of diabetes, DR has a global prevalence of 20%−35% in diabetic patients.^2^, ^3^ The global number of adults with DR is projected to rise significantly, increase by 25.9% to 129.84 million by 2030 and by 55.6% to 160.50 million by 2045. This substantial increase will pose a significant burden on society and the economy.^3^

DR is an irreversible blinding eye disease that is easy to prevent but difficult to treat. Early Treatment of Diabetic Retinopathy Study grading system is generally adopted to classify DR into non‐proliferative diabetic retinopathy (NPDR) and a more advanced stage, proliferative diabetic retinopathy (PDR).^4^ A delay in proper management can lead to irreversible vision loss; therefore, early detection and intervention in the initial stages of DR are of vital importance.^5^ Nevertheless, besides general systemic glycaemic control during the whole course and vitrectomy surgery for late‐stage patients, current treatments of DR are relatively limited. Laser photocoagulation only helps in halting the progression of moderate visual loss but does not improve visual acuity. Intravitreal anti‐vascular endothelial growth factor (VEGF) injection, mainly aimed at microvascular complications in DR, does not achieve optimal outcomes in every patient. Therefore, a complete understanding of disease pathophysiology is needed to develop new therapeutic approaches for DR, especially in the initial stages.^6^, ^7^

The mechanisms of DR involve molecular pathways, including inflammation, oxidative stress, renin–angiotensin system activation, VEGF, etc.^1^, ^8^ Previous research has explored the pathophysiology of DR extensively; however, traditional techniques such as microarrays and bulk RNA sequencing that assess transcripts from the whole tissue cannot distinguish relevant signals from heterogeneous cell populations.^9^ Consequently, new approaches that can account for cellular heterogeneity are required to investigate the role of the differentially expressed genes (DEGs) in DR pathogenesis.

Single‐cell RNA sequencing (scRNA‐seq), a novel technology invented by Tang et al.,^10^ allows high‐throughput molecular profiling of cells across different modalities. This approach has advanced rapidly with diverse methods and platforms such as Smart‐seq, Drop‐seq, Microwell‐seq, etc.,^11^ providing unprecedentedly deep insights into the complex processes involved in development, physiology and disease.^12^, ^13^, ^14^, ^15^, ^16^, ^17^, ^18^, ^19^, ^20^, ^21^, ^22^ In 2015, Macosko et al.^23^ first applied scRNA‐seq in ophthalmology to create a molecular atlas of gene expression for known retinal cell classes and novel candidate cell subtypes in the mouse retina. Subsequently, scRNA‐seq has become a tool in exploring a variety of eye diseases.^24^, ^25^, ^26^, ^27^, ^28^, ^29^, ^30^ In 2020, Van Hove et al.^31^ first attempted to employ scRNA‐seq to reveal the molecular and cellular changes in DR using Akimba mice model. Since then, an increasing number of studies have investigated DR using this method. As shown in Table 1, the application of scRNA‐seq to the study of DR has seen significant advancements.^31^, ^32^, ^33^, ^34^, ^35^, ^36^, ^37^, ^38^, ^39^, ^40^, ^41^, ^42^, ^43^, ^44^, ^45^, ^46^, ^47^, ^48^, ^49^, ^50^, ^51^, ^52^, ^53^, ^54^, ^55^, ^56^, ^57^, ^58^, ^59^, ^60^, ^61^

**TABLE 1:** Synoptic review of studies exploring diabetic retinopathy (DR) using single‐cell RNA sequencing.

Species	Sample	DR type	Retinal phenotypes	Number of cells	Type of cells	Keynote	Deposited datasets	Reference
Human	PBMC	Type 2 diabetic macular oedema patients (*n* = 4) versus healthy individuals (*n* = 4)	Type 2 diabetic macular oedema	32 731 (DR); 24 919 (control)	Monocytes, T cells, NK cells, B cells, dendritic cells	Immune landscape of diabetic macular oedema patients and identification of a pro‐inflammatory CD14++ monocytes subset	PRJCA006081	Ma et al.^32^
		Type 1 DR patients (*n* = 3) versus non‐DR diabetic patients (*n* = 3) and healthy individuals (*n* = 5)	Type 1 DR	20 453 (DR); 25 451 (NDR); 25 641 (healthy control)	T cells, NK cells, classical monocytes, non‐classical monocytes, B cells, conventional type 2 dendritic cells, basophils, plasmacytoid dendritic cells, platelets	Circulating immune cells show unique transcriptomic patterns in type 1 DR with JUND participating as a key factor	GSE248284; HRA000150	Liao et al.^33^
	FVM, retina, PBMC	PDR patients (FVM, *n* = 5) versus healthy individuals (retina and PBMCs)	PDR	6894 (FVM)	Microglia, monocyte, macrophage, fibroblast, pericyte, ECs, dendrite cell, T lymphocyte	Novel microglia phenotypes participate in FVM formation in PDR	GSE165784; EGAS00001004561; GSE175499	Hu et al.^34^
	FVM	PDR patients (*n* = 5)	PDR	7971	Macrophages, monocytes, B cells, CD8+ T cells, fibroblasts	Key genes and cellular components for PDR	GSE165784	Gao et al.^35^
		PDR patients (*n* = 2)	PDR	3584	Microglia, fibroblasts, ECs, dendritic cells, pericytes, neutrophils, macrophages, T cells, B cells	Vasculopathy of PDR and Alzheimer‘s disease share a common APP‐related signalling pathway	GSE165784	Xu et al.^36^
		PDR patients (*n* = 4)	PDR	N/A	Immune cells, stromal cells, ECs	Characterising molecular profiles of main cell populations in FVMs and the regulation of its formation	GSE245561	Corano‐Scheri et al.^37^
Monkey	Retina	Spontaneous type 2 diabetes (*n* = 1) versus non‐diabetic age matched (*n* = 1)	N/A	1555 (diabetes); 8708 (control)	Rod, cone, BCs, amacrine cells, Müller cells, microglia	Cell‐specific molecular changes and cell‒cell interactome of retina modulating microglia activation under DR	GSE168908	Xiao et al.^38^
Mice	Retina	12‐week‐old Akimba (*n* = 4) versus wild type (*n* = 2)	Severe retinal vascular pathology, intraretinal oedema, photoreceptor degeneration	5738 (Akimba); 3736 (control)	Rods, cones, BCs, amacrine cells, macroglia (Müller glia, astrocytes), immune cells, ECs, pericytes, fibroblasts	Molecular pathways underlying inflammatory, metabolic and oxidative stress‐mediated changes in Akimba DR mouse	E‐MTAB‐9061	Van Hove et al.^31^
				N/A	Rods, cones, BCs, amacrine cells, macroglia, immune cells, ECs, pericytes	High glucose exposure induces premature senescence in retinal ECs	E‐MTAB‐9061	Bertelli et al.^39^
		8‐month‐old db/db (*n* = 3) versus db/m (*n* = 3)	Inflammation, apoptosis, acellular capillaries, neuronal dysfunction	51 558 (db/db); 34 010 (control)	Rods, cones, cone BCs, rod BCs, Müller glia, amacrine cells, RGCs, microglia, ECs, pericytes, horizontal cells	Transcriptional landscape and heterogeneity of retinal cells in type 2 diabetic mice	PRJNA653629	Niu et al.^40^
				N/A		Single‐cell atlas of alternative transcription start sites of healthy and diabetic retina	PRJNA653629	Mao et al.^41^
		13‐week‐old db/db (*n* = 2) versus wild type (*n* = 2)	Neuron microstructure alterations, no obvious microvascular pathology	9107 (db/db); 7549 (control)	Rods, cones, cone BCs, rod BCs, Müller glia, amacrine cells, microglia	Cross‐species retinal cell atlas and early alterations in type 2 diabetes mice	Available on request	Chen et al.^42^
		21‐week‐old db/db (*n* = 8) versus wild type (*n* = 8)	Thinner retina, acellular capillaries, abnormal vessels, capillary leakage	8048 (db/db); 6369 (control)	ECs, pericytes	Retinal pericytes share similar gene expression and molecular transitions with renal mesangial cells under diabetes	GSE204880	Xu et al.^43^
		20‐week‐old db/db (*n* = 2) versus db/m (*n* = 2)	Impaired visual function, acellular capillaries	18 376 (total)	ECs, pericytes, photoreceptors, microglia, Müller cells, neurons	A new diabetes‐specific retinal EC population and a negative feedback regulation pathway reducing endothelial dysfunction via ACER2	Available on request	Yao et al.^44^
		16‐week‐old db/db (*n* = 2) versus db/m (*n* = 2)	Thinner retina, photoreceptor injury and apoptosis, impaired visual function	N/A	Rods, cones, cone BCs, rod BCs, amacrine cell, microglia, Müller glia, immune cells (monocytes, macrophages), pericytes, ECs	WNT‐inhibitory factor 1‐mediated glycolysis protects photoreceptor cells in DR	Available on request	Chen et al.^45^
		db/db versus db/m	Thinner retina, acellular capillaries	51 327 (total)	Rods, cones, cone BCs, Müller cells, horizontal cells, epithelial, ECs	p53 accelerates endothelial cell senescence in DR	Available on request	Cheng et al.^46^
	Retina	STZ induced (*n* = 3) versus untreated (*n* = 3)	Inflammation, impaired visual function	8239 (STZ); 6185 (control)	Rods, cones, BCs, amacrine cells, RGCs, Müller glia, astrocytes, microglia, ECs, pericytes, RPEs	Pathological alterations of gene expression in STZ‐induced DR	GSE178121	Sun et al.^47^
				13 336 (STZ); 8252 (control)	Rods, cones, BCs, anaplastic cells, Müller cells, astrocytes, microglia, ECs, pericytes, RPEs	Changes in retinal cell subpopulation levels and the pathways involved in DR	GSE178121	Zhang et al.^48^
				7851 (STZ); 6126 (control)	Rods, cones, BCs, amacrine cells, RGCs, Müller cells, astrocytes, microglia, vascular ECs, pericytes, RPEs	Activated retinal microglia contribute to inflammation in STZ‐induced early DR	GSE178121	Lv et al.^49^
		STZ induced (*n* = 5) versus sham (*n* = 5)	Inflammation, vascular dysfunction	12 003 (STZ); 19 253 (control)	Rods, cones, BCs, amacrine cells, RGCs, Müller cells, microglia, ECs, pericytes	Microglia–endothelial intercellular communication networks and CSF1/CSF1R dysregulation in PDR	Available on request	Ben et al.^50^
	Retina	OIR (*n* = 6) versus normoxia (*n* = 6)	Neovascularisation	17 701 (total)	N/A	A specific RIP3+ subpopulation of microglia promotes retinopathy through a hypoxia‐triggered necroptotic mechanism	GSE152928	He et al.^51^
		OIR (*n* = 3) versus normoxia (*n* = 2)	Neovascularisation	N/A	Rods, cones, BCs, Müller glia, astrocytes, amacrine cells, RGCs, immune cells, ECs, pericytes,, horizontal cells	Pathological angiogenesis in retinopathy engages cellular senescence especially in vascular units	GSE150703	Crespo‐Garcia et al.^52^
		OIR (*n* = 8) versus normoxia (*n* = 4)		30 456 (total)	Conets, BCs, amacrine cells, RGCs, Müller glia, astrocytes, immune cells, horizontal cells, ECs, pericytes, microglia	STING highly expressed in retinal vessels mediates endothelial inflammatory injury	GSE150703	Wen et al.^53^
		OIR (*n* = 8) versus normoxia (*n* = 4)		31 271 (total)	N/A	A Tsp‐1+ microglia subpopulation attenuates retinal neovascularisation	GSE150703	Luo et al.^54^
		OIR (*n* = 8) versus normoxia (*n* = 4)		N/A	Rods, cones, bipolar cells, amacrine cells, Müller glia, astrocytes, RGCs, ECs, pericytes, microglia/macrophages, horizontal cells, fibroblasts	Increased GNAI2 in retinal EC is pivotal in pathological angiogenesis in DR	GSE150703	Bai et al.^60^
		OIR (*n* = 16) versus normoxia (*n* = 16)	Neovascularisation	Only CD11bF4/80: 1259 (OIR); 1449 (control)	Rods, cones, BCs, Müller glia, immune cells, ECs, pericytes, microglia, monocytes, macrophages	Several unique microglia subtypes associated with proliferative retinopathy	GSE199792	Liu et al.^55^
		OIR (*n* = 2) versus normoxia (*n* = 1)	Neovascularisation	N/A	Rods, cones, BCs, Müller glia, microglia, monocytes/macrophage, amacrine cells/horizontal cells, RGCs, ECs, pericytes, RPEs, erythrocytes	Microglia‐derived Spp1 promotes pathological retinal neovascularisation	PRJNA864092	Bai et al.^56^
		OIR (*n* = 2) versus normoxia (*n* = 2)	Neovascularisation	37 036 (OIR); 39 128 (control)	Photoreceptors, BCs, amacrine cells, RGCs, Müller glia, microglia, ECs, pericytes, RPEs, erythroid cells	A pericyte type uniquely expressing abundant Col1a1 is associated with capillary dysfunction	N/A	Xia et al.^57^
Rats	Retina	STZ induced (*n* = 3) versus sham (*n* = 2)	N/A	24 837 (STZ); 11 073 (control)	Rods, cones, BCs, horizontal cells, amacrine cells, Müller glia, microglia, ECs, pericytes	Müller subtypes and inner blood–retinal barrier regulatory network in early DR	GSE209872	Wang et al.^58^
					Rods, cones, BCs, horizontal cells, amacrine cells, Müller glia, macrophages, microglia, ECs, pericytes	Unique microglia subtypes and their polarisation characteristics involved in early DR	GSE209872	Wang et al.^59^
				N/A	Rods, cones, BCs, horizontal cells, amacrine cells, macroglia, macrophages, microglia, ECs, pericytes	Increased GNAI2 in retinal EC is pivotal in pathological angiogenesis in DR	GSE209872	Bai et al.^60^
Zebrafish	Retina	Glucose‐treated pdx1+/‒ mutants (*n* = 6) versus pdx1+/‒ mutants (*n* = 6) and wild type (*n* = 6)	Microvascular defects, cone morphologic alteration	3915 (glucose‐treated pdx1/‒ mutants); 2994 (pdx1/‒ mutants); 8142 (wild type)	Photoreceptors, BCs, amacrine cells, RGCs, Müller glia, macrophage/microglia, ECs	Progressively decreased HCN1 channels results in cone morphological defects in DR	Available on request	Han et al.^61^

Abbreviations: APP, amyloid‐beta precursor protein; BC, bipolar cell; EC, endothelial cell; FVM, fibrovascular membrane; NDR, non‐diabetic retinopathy; NK, natural killer; OIR, oxygen‐induced retinopathy; PBMC, peripheral blood mononuclear cell; PDR, proliferate diabetic retinopathy; RGC, retinal ganglion cell; RPE, retinal pigment epithelial; STZ, streptozotocin.

**FIGURE 1:**
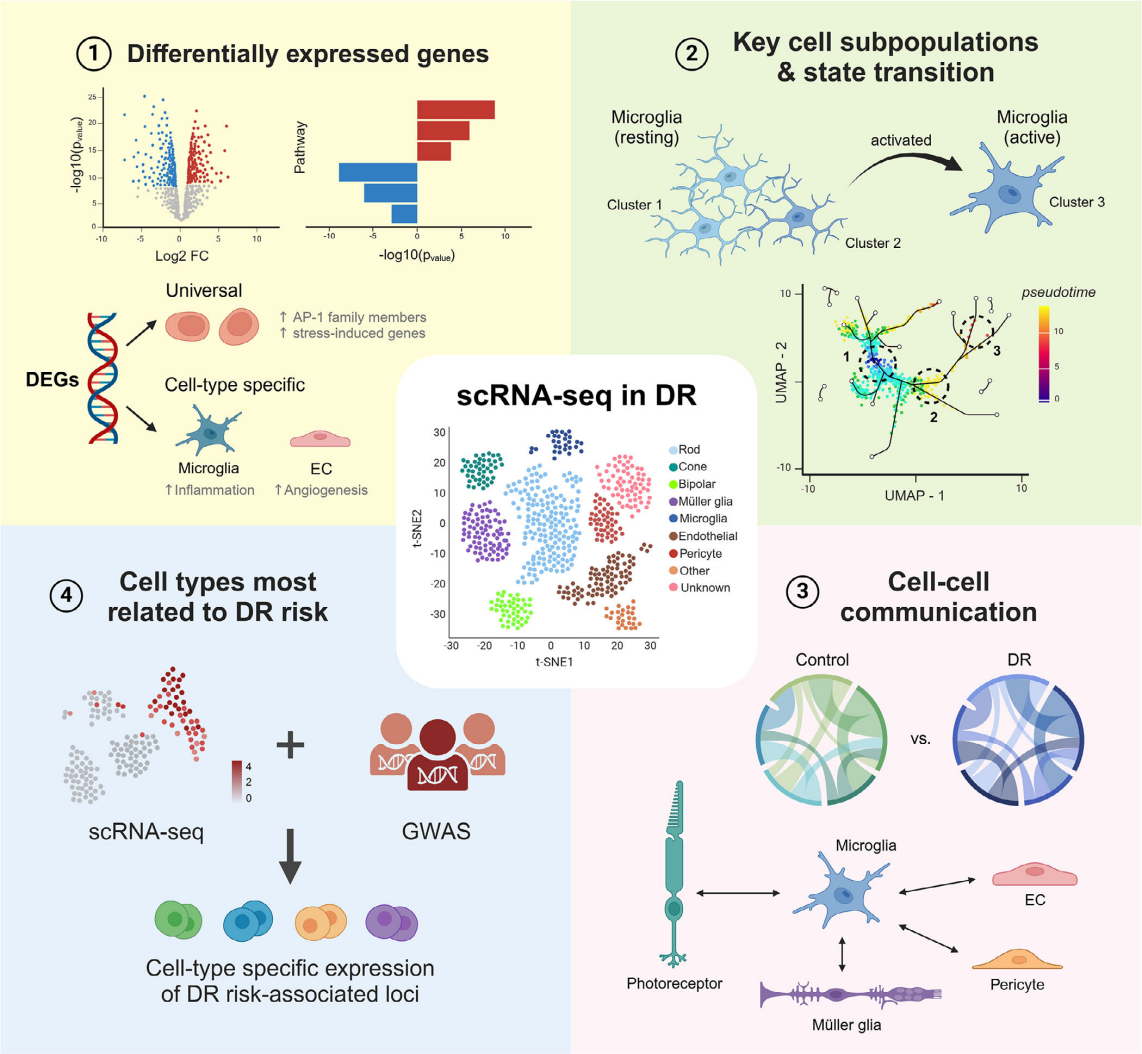
Overview of single‐cell RNA sequencing (scRNA‐seq) application in diabetic retinopathy research. The application of scRNA‐seq methodologies to the study of diabetic retinopathy (DR) highlights four key areas of focus: (1) the detection of differentially expressed genes (DEGs), (2) the identification of key cell subpopulations and their state transition, (3) the exploration of cell‒cell communication networks within DR and (4) the correlation of cell types with genetic risk loci for DR by integration with genome‐wide association studies (GWAS). EC, endothelial cell.

This review summarises the specific application of scRNA‐seq in DR from four perspectives (Figure 1). First, we discuss the DEGs between the DR and control groups, including universally changed and cell‐type‐specific DEGs. We subsequently enumerate several key cell subpopulations and their state transitions during DR. The known roles of cell‒cell communication in the pathogenesis of the disease are also discussed. Finally, we outline studies that combined scRNA‐seq with genome‐wide association studies (GWAS) to identify the cell types most closely linked to the genetic loci associated with DR risk. Given the unique strengths of scRNA‐seq, we anticipate that future research using single‐cell omics to will delve deeper into the mechanisms underlying DR and develop novel effective targeted therapeutic approaches.

## 2 IDENTIFICATION OF DEGs IN DR BY scRNA‐seq

The retina is a complex and delicate tissue composed of various cell types, including photoreceptors (rods and cones), bipolar cells, amacrine cells, horizontal cells, retinal ganglion cells, astrocytes, Müller glia, microglia, endothelial cells (ECs) and pericytes. Traditional bulk RNA sequencing methods can only detect the average gene expression in the whole tissue wherein cell‐type‐specific differences in gene expression are easily to be masked unless the differences are sufficiently prominent. scRNA‐seq analyses the transcriptome at the single‐cell level, thus allowing the depiction of gene expression distributed in specific cell populations and enabling comparison between different conditions to overcome the limitation of bulk sequencing, thereby facilitates the multi‐dimensional and in‐depth analysis of pathological mechanisms underlying DR to develop novel therapeutic approaches.

Previous studies have demonstrated that glial cells produce various inflammatory factors in the early stages of DR, and that continuous chronic inflammation may further contribute to neovascularisation in the advanced stage of DR.^62^, ^63^ However, the specific role of microglia in the pathogenesis of DR is still not completely understood. Lv et al.^49^ generated the single‐cell transcriptomic profiles of streptozotocin (STZ)‐induced diabetic mice retina to identify the main cell types producing pro‐inflammatory cytokines at the early stage of DR and identified microglia as the major source of interleukin (IL)‐1β and tumour necrosis factor (TNF). To address the function of microglia in late DR, Liu et al.^55^ and Bai et al.^56^ both performed scRNA‐seq in mice with oxygen‐induced retinopathy (OIR), a commonly used model for proliferative retinopathy. Igf1 and Spp1, respectively, were observed to be upregulated in OIR microglia compared to control microglia. Suppression of these two DEGs significantly attenuated the pathological retinal neovascularisation in OIR mice, indicating robust angiogenic effects of these genes. Consistently, clinical research has observed increased vitreous levels of insulin‐like growth factor 1 (IGF‐1) and secreted phosphoprotein 1 (SPP1) in patients with DR than the non‐DR patients.^64^, ^65^, ^66^, ^67^, ^68^ These results provide strong evidence for exploiting the inhibition of *IGF1* or *SPP1* as a prospective therapy for DR.

*RLBP1* encodes cellular retinaldehyde‐binding protein, which is vital for the retinal visual cycle.^69^ It has been widely studied in the context of autosomal recessive retinal diseases but had been scarcely associated with DR.^70^ Niu et al.^40^ discovered an opposing trend of Rlbp1 expression change between Müller glia and other retinal cells in db/db mice via scRNA‐seq. While upregulated in other cell types, Rlbp1 was shown to be downregulated in Müller glia, which may explain why previous bulk measurements did not distinguish its alteration in DR. Upon selective overexpression of Rlbp1 in diabetic Müller glia, the gliosis was reduced and the retinal capillaries and neurons were protected, suggesting the supplementation of Rlbp1 as a promising approach to mitigate DR‐associated dysfunction.

**FIGURE 2:**
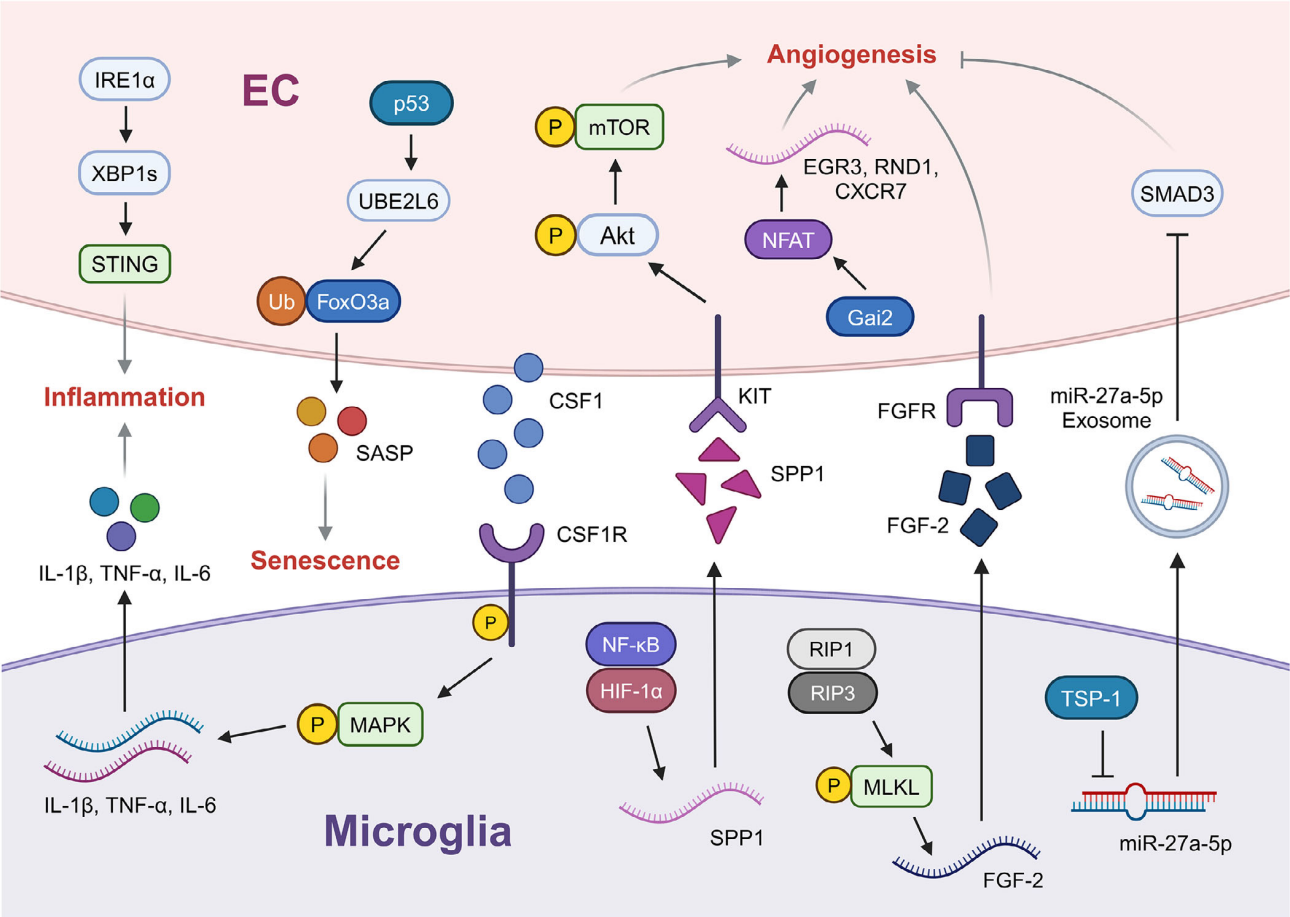
Signalling pathways in diabetic retinopathy revealed by single‐cell RNA sequencing (scRNA‐seq). The figure delineates the diabetic retinopathy (DR)‐related signalling pathways and the interactions between microglia and endothelial cells (ECs) that have been elucidated through scRNA‐seq. These pathways are catalogued to provide a comprehensive view of the cellular communication and signalling networks implicated in DR pathogenesis. Akt, AKT serine/threonine kinase 1; CSF, colony stimulating factor; CXCR7, C‐X‐C motif chemokine receptor 7; EGR3, early growth response 3; FGF‐2, fibroblast growth factor 2; FoxO3a, forkhead box O3; Gai2, G protein subunit alpha i2; IL, interleukin; IRE1, inositol‐requiring enzyme 1; KIT, KIT proto‐oncogene, receptor tyrosine kinase; MAPK, mitogen‐activated protein kinase; MLKL, mixed lineage kinase domain like pseudokinase; mTOR, mechanistic target of rapamycin kinase; NFAT, nuclear factor of activated T cells; RIP, receptor interacting serine/threonine kinase; RND1, Rho family GTPase 1; SASP, senescence‐associated secretory phenotype; SMAD, small mothers against decapentaplegic; SPP1, secreted phosphoprotein 1; STING, stimulator of interferon response cGAMP interactor; TNF, tumour necrosis factor; TSP‐1, thrombospondin 1; UBE2L6, ubiquitin conjugating enzyme E2 L6; XBP1, X‐box binding protein 1.

The activation of ECs triggered by VEGF and other stimuli has been viewed as a pivotal process in neovascularisation in DR. However, the downstream mediators of angiogenesis remain largely unknown. Bai et al.^60^ analysed the scRNA‐seq data from STZ‐induced diabetic mice and mice with OIR, and found that G protein subunit alpha i2 (Gαi2) was ubiquitously expressed across all cell types but significantly elevated in ECs, suggesting its potential role in ECs. Subsequent experiments validated the role of Gαi2 in activating the transcription factor NFAT to promote retinal pathological angiogenesis in DR mice (Figure 2).

Although most genes were deregulated only in a certain type of retinal cells, several were altered in multiple cell types.^47^ Niu et al.^40^ observed the upregulation of Fos, Madd and Pttg1 in 8‐month db/db mice, while in a study by Sun et al.,^47^ the top five most commonly deregulated genes in various retinal cell types were Cirbp, Mt1, Rbm3, Hmgb2 and Mt2. These genes are characterised as stress‐induced and associated with inflammation, oxidative stress response, neuron death and apoptosis. Liao et al.^33^ collected peripheral blood mononuclear cells (PBMCs) from patients with type 1 diabetes to construct a single‐cell atlas of circulating immune cells involved in DR and found that the expression of AP‐1 family members was significantly upregulated and enriched in pro‐inflammatory pathways in T cells, B cells and monocytes of patients with DR compared to non‐DR or healthy individuals. Targeting such universal DEGs may offer more comprehensive and powerful therapeutic efficacy by targeting multiple retinal cells.

In summary, scRNA‐seq enables more sensitive and elaborate detection of gene expression changes in specific cell types. Here, we summarised the DEGs discovered by scRNA‐seq and verified in previous studies, as well as their corresponding functions in DR (Table 2). Recognition of these DEGs facilitates our understanding of the molecular pathways underlying DR and further promotes the development of new targeted therapies.

**TABLE 2:** List of potential key regulatory genes identified by single‐cell RNA sequencing in diabetic retinopathy (DR).

Sample	DEGs	Changes in DR	Detected cell types	Functions	Reference
Retina	*Hcn1*	Down	Cone	Cone morphologic integrity	Han et al.^61^
	*Rlbp1*	Down	Müller glia	Component of visual cycle; mitigate neurovascular degeneration	Niu et al.^40^
	*S100a1/6/10/11/13/16, Gfap*	Up	Macroglia	Reactive gliosis markers	Van Hove et al.^31^
	*Col1a1*	Up	Pericyte	Pathological angiogenesis	Xia et al.^57^
	*Tp53*	Up	EC	Cellular senescence	Bertelli et al.^39^
					Cheng et al.^46^
	*Acer2*	Up	EC	Sphingolipid metabolism; regulate EC barrier function	Yao et al.^44^
	*Vcam‐1*	Up	EC	Inflammation and cell adhesion	
	*Col1a1*	Up	EC	Fibrosis; FVM formation	Crespo‐Garcia et al.^52^
	*Tmem173*	Up	EC	Mediate endothelial inflammatory and injury	Wen et al.^53^
	*Gnai2*	Up	EC	EC activation and angiogenesis	Bai et al.^60^
	*Csf1*	Up	EC	Elicit microglia differentiation and secretion of inflammatory factors; pro‐angiogenesis	Ben et al.^50^
	*Csf1r*	Up	Microglia		
	*Il1b, tnf*	Up	Microglia	Inflammation	Lv et al.^49^
	*Rip3, Mlkl*	Up	Microglia	Necroptosis; mediate FGF‐2 to induce retinal angiogenesis	He et al.^51^
	*Tsp‐1*	Up	Microglia	Inhibit angiogenesis	Luo et al.^54^
	*Pkm*	Up	Microglia	Glycolysis	Liu et al.^55^
	*Igf1*	Up	Microglia	Pro‐angiogenesis	
	*Spp1*	Up	Microglia	Mediate microglia–EC communication; promote retinal neovascularisation	Bai et al.^56^
FVM	*FN1*	Up (FVM vs. healthy retina)	Microglia	Fibrosis; FVM formation	Hu et al.^34^
PBMC	*JUND*	Up	Universal	Regulate PBMC‐mediated endothelial dysfunction	Liao et al.^33^

Abbreviations: DEG, differentially expressed gene; EC, endothelial cell; FGF‐2, fibroblast growth factor 2; FVM, fibrovascular membrane; PBMC, peripheral blood mononuclear cell.

## 3 KEY CELL SUBPOPULATIONS AND STATE TRANSITION IN DR

**FIGURE 3:**
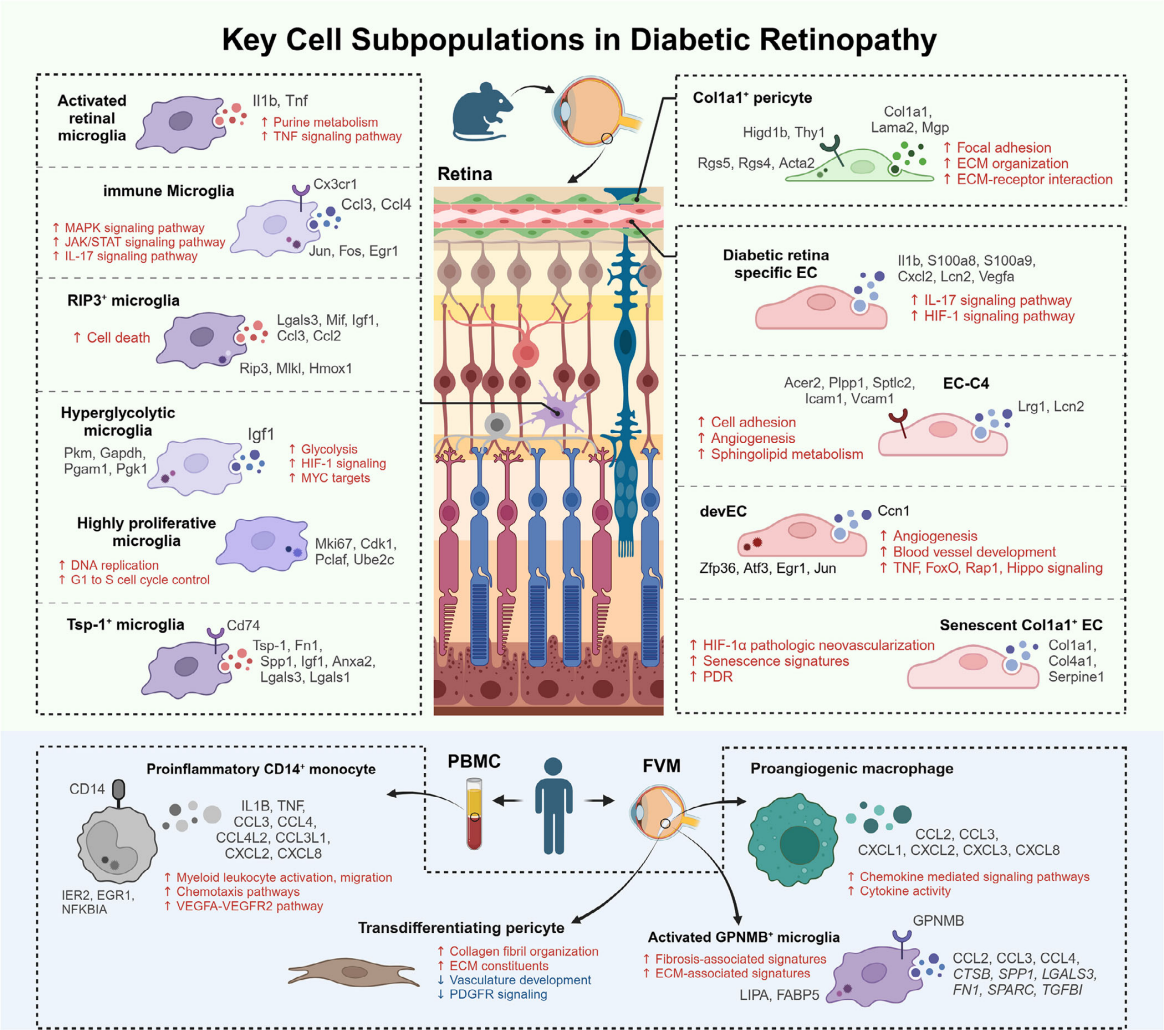
Identification of key cell subpopulations in diabetic retinopathy (DR) by single‐cell RNA sequencing (scRNA‐seq). The figure illustrates the key cell subpopulations detected in DR from animal models (upper panel) and human samples (lower panel). These subpopulations are highlighted to delineate the cellular heterogeneity during the progression of DR, providing insights into the distinct roles of different cell clusters in the pathophysiology of the disease. Cell names marked in bold black indicate key subpopulations associated with DR as reported in single‐cell sequencing studies. These names are excerpted from the literature. Red arrows and fonts indicate the enriched upregulated pathways in the subpopulation. Blue arrows and fonts indicate the enriched downregulated pathways in the subpopulation. Black fonts indicate the unique expressed intracellular/secreted protein or membrane receptor in the subpopulation. EC, endothelial cell; FVM, fibrovascular membrane; PBMC, peripheral blood mononuclear cell.

Cell heterogeneity in pathological conditions is informative for unveiling the mechanisms of the disease. Unlike traditional cell characterisation methods that use known markers to identify a limited number of cell types, scRNA‐seq allows for high‐dimensional sequencing of cells, grouping them into various populations based on their transcriptional signatures. This enables the discovery of previously unknown cell subtypes that are crucial to understanding the disease. Based on the description of static cell features, scRNA‐seq leverages advanced algorithms and computational methods to assign each cell a specific position along a continuous trajectory as ‘pseudo‐time’ to infer the dynamic processes of cell transition between various states.^71^ The identification of unique clusters and their transformation in DR favours better inquiry into the pathogenesis of the disease and latent remedies (Figure 3).

### 3.1 Microglia

Microglia play important roles of inflammation in the early stage of DR. Previous studies has reported the microglia phenotypic switch in DR as from the alternative ‘M2’ (anti‐inflammatory) state to the classical ‘M1’ (pro‐inflammatory) state.^72^ This simple dichotomous paradigm, however, is inadequate to fully describe the complicated biology of microglia activation in vivo.^73^ The emergence and introduction of scRNA‐seq have revolutionised the profiling of individual cells with high‐throughput datasets, thereby achieving a more precise and multifaceted characterisation of microglia subpopulations in DR.^74^

Activated microglia secrete various pro‐inflammatory cytokines and chemokines that cause chronic inflammation. The morphology and location of these cells are altered in DR.^75^, ^76^, ^77^ However, traditional methods are inefficient in characterising these activated retinal microglia. Using scRNA‐seq, Lv et al.^49^ identified a subcluster of microglia within the retina of STZ‐induced diabetic mice characterised as the activated retinal microglia that predominantly expressed IL‐1β and TNF in the retina. Kyoto Encyclopedia of Genes and Genomes analysis suggested that metabolic and inflammation‐related pathways were enriched in this cluster and further integrated multi‐omics analysis revealed that this activated microglial subpopulation has a metabolic bias favouring glycolysis, purine metabolism and triacylglycerol synthesis, but less tricarboxylic acid. Ben et al.^50^ also identified a microglial cluster that was enriched in the STZ‐induced DR group manifesting immunoregulatory signatures (named immune microglia). The cluster was placed at the end of the pseudo‐time trajectory, along which the mitogen‐activated protein kinase (MAPK), JAK/STAT and IL‐17 signalling pathway were significantly upregulated, indicating its pro‐inflammatory function as the activated state of microglia. Targeting activated microglia may reduce the retinal inflammation, thereby retarding the early DR progression.

In addition to inflammation, the activation and accumulation of microglia are also correlated with pathogenic angiogenesis in DR.^75^, ^78^ Although studies using OIR models have reported a switch from M1 polarised microglia in the early stage of neovascularisation towards M2 polarisation in the late phase,^79^ the specific phenotype of microglia exerting promotional or inhibitory effects is relatively unclear. He et al.^51^ performed single‐cell RNA profiling on the retinal microglia of OIR mice and identified a subpopulation that highly expressed the necroptosis‐related genes *Rip3* and *Mlkl*. Hypoxia‐triggered necroptotic microglia released fibroblast growth factor 2 (FGF‐2), which acts on ECs to induce retinal neovascularisation (Figure 2). Liu et al.^55^ also mapped microglial populations in OIR retina and found two clusters that appeared primarily in the OIR group and expressed high levels of microglial activation markers. One subset was characterised by a high level of glycolytic genes and the proangiogenic gene *Igf1*, whereas the other manifested significantly upregulated proliferation‐related genes and high self‐renewal capacity. RNA velocity analysis confirmed that the activated retinal microglia were the precursor cells driving the expansion and metabolic activation of inflammatory microglia during OIR. Intriguingly, in the study by Luo et al.,^54^ although a retinal microglial population expressing activated markers, such as *Spp1* and *Igf1*, was significantly enriched in the OIR group; among them, there was a small subset expressing *Tsp‐1* and *Fn1*, despite the limited absolute ratio. These microglia attenuated retinal neovascularisation via the Tsp‐1/miR‐27a‐5p exosome/Smad3 axis. These results demonstrate the complexity of microglia in the regulation of angiogenesis in DR. Therefore, a comprehensive description of microglia subtypes using scRNA‐seq will be valuable for exploring the mechanisms of neovascularisation in DR.

scRNA‐seq has also illuminated the presence of retina‐derived microglial in the fibrovascular membranes (FVMs) of patients with proliferative DR, suggesting a potential role in FVMs formation. These FVMs that grow between the retina and posterior hyaloids of the vitreous can cause tractional retinal detachment and subsequent vision loss in patients with proliferative DR. Despite previous studies investigating the formation of FVM,^80^, ^81^, ^82^, ^83^ its cellular composition has not been clarified owing to the limitations of conventional methods. Hu et al.^34^ generated a comprehensive cell atlas of FVM from patients with proliferative DR and regarded microglia as a major cell population. Among these, the GPNMB+ microglial subtype exhibits both pro‐fibrotic and fibrogenic properties. Pseudo‐time analysis further revealed that this profibrotic cluster was the major activation state of microglia involved in the formation of FVM and differentiated from retina‐resident microglia rather than from monocyte‐derived macrophages. Besides, *SPP1* was observed significantly enriched in this activated microglial cluster compared with other cell types, in accordance with the findings from animal models by Bai et al.^56^ However, in a study by Corano‐Scheri et al.,^37^ a cluster of macrophages showing high expression of pro‐angiogenetic chemokines, such as CXCL1, CXCL2 and CCL2, was identified, instead of microglia. The authors claimed that they were unable to replicate the conclusion of Hu et al.^34^ as they regarded the majority of immune cells in FVM as macrophages rather than microglia. This controversy might be attributed to the discrepancy in the markers that identify cell clusters. However, further studies are required to reach more rigorous conclusions.

Collectively, the characterisation of activated microglia that promote inflammation or angiogenesis in DR offers new insights into the pathogenesis of the disease and helps develop targeted therapies that delay DR progression.

### 3.2 Endothelial cell

EC dysfunction leads to capillary dilatation, leakage, rupture and neovascularisation during the process of DR.^1^ Studies have validated that ECs enhance inflammatory molecules, such as intercellular adhesion molecule 1 (ICAM‐1), to stimulate leukocyte adherence and interact with VEGF, causing increased vascular permeability and angiogenesis.^62^, ^84^ However, it remains essential to explore which specific populations of ECs are more susceptible to hyperglycaemia and are likely to induce inflammation and neovascularisation.

Sun et al.^47^ identified a subpopulation of EC that specifically expressed S100a8, S100a9 and Il1β, and named it as diabetic retina endothelial cells (DRECs) following a dissection of single‐cell transcriptomics data of retinas from STZ‐induced diabetic mice and controls. This subpopulation exhibited a significant enrichment of inflammation‐associated pathways, such as IL‐17, TNF and necrosis factor‐kappa B (NF‐κB) signalling pathways. Moreover, *Hif1a* and *Vegfa*, the critical genes of hypoxia inducible factor (HIF)‐1 signalling that were confirmed to be important for DR, were also upregulated in DRECs only. Yao et al.^44^ also identified a unique EC cluster only present in the diabetic retina of db/db mice, highly expressing *Icam‐1* and *Vcam‐1*. Apart from the inflammation and cell migration pathways, this distinct EC cluster uniquely expressed the cell markers of ceramidases involved in sphingolipid metabolism, including *Acer2* and *Plpp1*. Subsequent experiments verified the pro‐angiogenic effect of *Acer2*, and the combination of ACER2 and Lucentis synergistically inhibited VEGF‐induced EC permeability. In the study by Ben et al.,^50^ ECs were classified into three groups and one cluster comprised a significantly increased proportion in the STZ‐induced DR group compared to the wild type. The cluster exhibited strong activation of vascular development and induction of inflammation‐related pathways, and was represented at the termination of the pseudo‐time trajectory as a vascular development state named devEC. Evidence above implies that some ECs may be more susceptible to the hyperglycaemic environment in DR.

Cellular senescence has been associated with the pathogenesis of DR.^85^ Single‐cell analysis of db/db mice, Akimba mice and mice with OIR all revealed the enrichment of senescent gene signatures in ECs, including the upregulation of the senescence biomarker p53.^39^, ^46^, ^52^ In the study by Crespo‐Garcia et al.,^52^ pathological vessels were found to engage pathways of cellular senescence in contrast to healthy ones, and inhibitors of the anti‐apoptotic protein BCL‐xL successfully suppressed pathological angiogenesis. Interestingly, single‐cell analysis revealed that a single cluster of ECs with senescence signatures and unique expression of *Col1a1* was no longer detected in the BCL‐xL‐inhibitor‐treated retinas, implying that the senolytic drug selectively eliminated senescent cells to suppresses neovascularisation. Notably, this Col1a1+ EC cluster harboured a transcriptomic signature orthologous to the senescent human retinal microvascular ECs profile, indicating the cross‐species applicability of this finding. The ability of senolytics to clear senescent cells without harming healthy tissues suggests their strong potential for treating DR.

In summary, among all the ECs, some are more vulnerable to hyperglycaemia with enriched pathways related to inflammation, angiogenesis and senescence during the process of DR. Eliminating such specific subpopulations may prevent vascular leakage and neovascularisation to retard the disease progression.

### 3.3 Other cells

In the retina, pericytes, as part of the vascular unit, maintain the integrity of the blood‐retina barrier and regulate vessel development and angiogenesis.^86^, ^87^, ^88^ Although the pericyte degeneration and death in the early phases of DR have been widely studied, studies focusing on their function in promoting neovascularisation in advanced DR are relatively few. Xia et al.^57^ created an atlas of single‐cell transcriptomes of retinas from mice with OIR and mice in room air. They identified a pericyte subpopulation that uniquely expressed high levels of *Col1a1* and other genes related to extracellular matrix remodelling. This subpopulation was significantly more abundant in OIR retinas compared to control retinas. Subsequent experiments verified that Col1a1 was increased in pericytes of the OIR retina, and its silencing suppressed ocular angiogenesis and pericyte‒myofibroblast transition during capillary remodelling. In another study by Corano‐Scheri et al.,^37^ an intermediate cluster expressing genes overlapping with both pericytes and myofibroblasts was identified, suggesting that cells transitioned from a pericyte origin towards a myofibroblastic identity, with an upregulated profibrotic molecule, *AEBP1*, that modulates the process. Considering that this transition may further aggravate fibrosis and contraction of FVMs resulting in tractional retinal detachment, *AEBP1* could serve as a potential therapeutic target for preventing scar tissue formation in DR.

In DR, photoreceptors are affected even before the vascular abnormalities develop, causing defects in visual function.^89^ However, the identification of which type of photoreceptors is more vulnerable to hyperglycaemia and the molecular mechanisms responsible for photoreceptor damage are incomplete. Han et al.^61^ developed a progressive DR model using pdx1+/‒ mutants and glucose treatment in adult zebrafish. As they progressed from wild‐type zebrafish to pdx1+/‒ mutants and then to glucose‐treated pdx1+/‒ mutants, the zebrafish exhibited increasing blood glucose levels and a shortening of cone segments. Single‐cell analysis showed that cones as the most vulnerable type of retinal neuron, underwent three distinct states along the pseudo‐time trajectory. In correspondence with progressive cone segment defects, a gradually declining expression pattern of *hcn1* was observed, indicating that *hcn1* reduction as a mechanism underlying cone defects in DR. Such identification of state‐specific gene profiles may further shed light on their potential role in the onset and progression of DR.

Apart from retina‐resident immune cells, circulating leukocytes may also participate in inflammation leading to DR.^62^, ^90^, ^91^ To better clarify the phenotypic and functional diversity of immune cells in DR, Ma et al.^32^ constructed the single‐cell RNA atlas of PBMCs from diabetic macular oedema patients and healthy individuals, wherein a subset of pro‐inflammatory CD14+ monocytes was identified. This cluster expressed increased levels of inflammatory cytokines and chemokines, such as TNF, IL‐1β and CCL3, as well as upregulated inflammatory pathways, demonstrating that it is a potential target cell subpopulation for rescuing immune dysregulation in DR.

Altogether, the application of scRNA‐seq has enabled a comprehensive characterisation of the cellular landscape within the diabetic retina. Identification of key subpopulations in DR and their transition states in DR provides more profound insights into the underlying pathogenic mechanisms. Targeting the unique markers or DEGs of these clusters may build an innovative and precise therapeutic avenue.

## 4 CELL‒CELL COMMUNICATION IN DR

**FIGURE 4:**
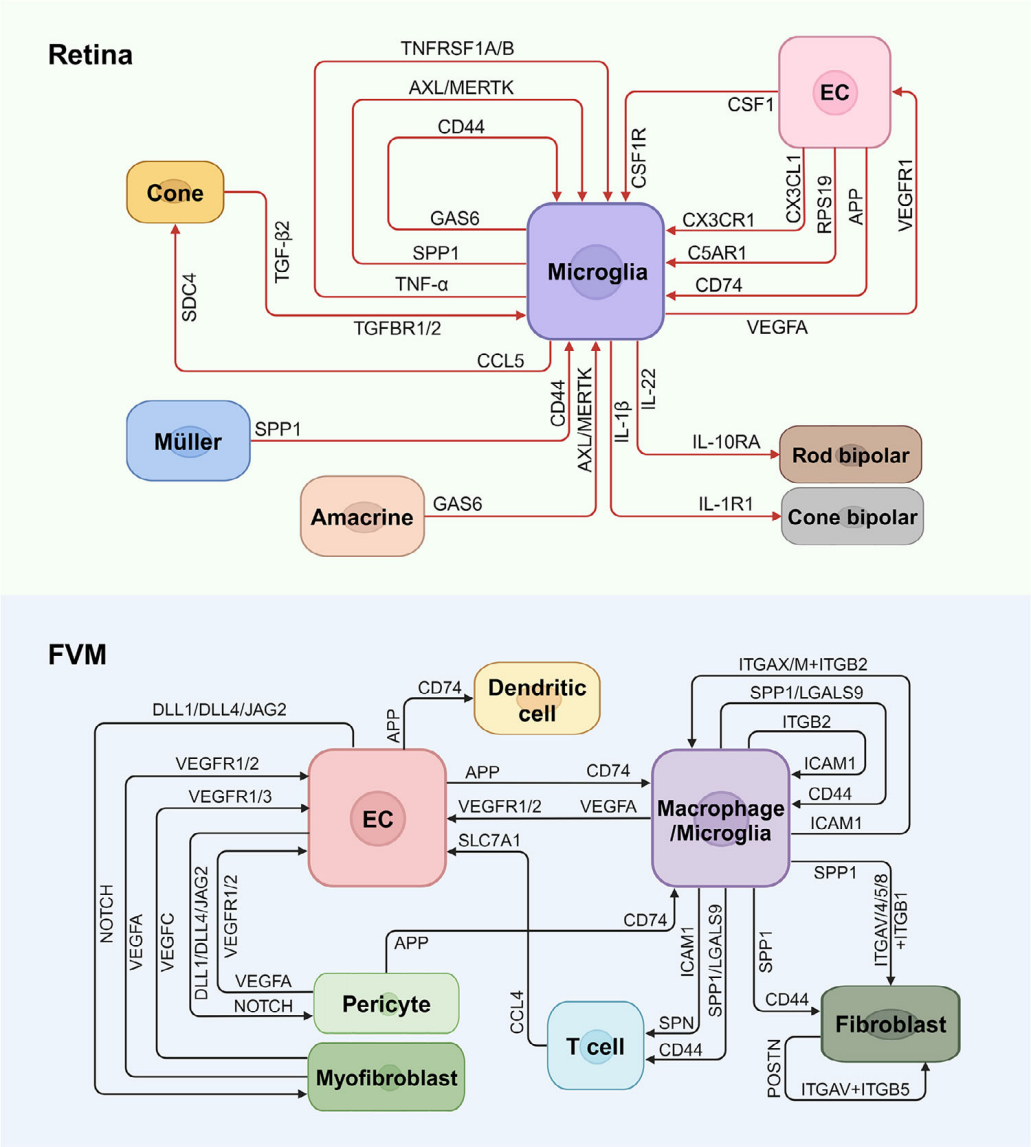
Cell‒cell communication in diabetic retinopathy revealed by single‐cell RNA sequencing (scRNA‐seq). The figure depicts the ligand‒receptor interactions implicated in diabetic retinopathy (DR), as detected by scRNA‐seq in previous research. Red lines indicate the upregulated interactions among retinal cell types in DR animal models (upper panel), while black lines represent the crosstalk among cells of fibrovascular membranes (FVMs) derived from human diabetic patients (lower panel). Each line labels with two proteins, with the protein closest to the arrowhead indicating the receptor expressed on the cell nearest to the arrow, and the protein closest to the arrowtail representing the ligand expressed on the cell nearest to the arrow. This visualisation elucidates the potential communication networks altered within the diabetic retinas and the intercellular crosstalk present within the FVMs, which may contribute to the pathogenesis of DR. EC, endothelial cell.

Organisms depend on intricate intercellular coordination for their proper functioning. Untangling the complex dynamics of physiological cell‒cell communication and its disturbance under pathological conditions enhances our understating of the disease and creates opportunities for the development of therapeutic interventions.^92^ With the expansion of protein‒protein interaction databases and advances in RNA sequencing technologies, ligand‒receptor pairs allow the inference of cell‒cell communication from gene expression data. Using computational analysis methods such as CellChat, iTALK and CellPhoneDB, scRNA‐seq offers distinctive advantages in identifying the cell types that produce proteins involved in intercellular communication. This allows for a more comprehensive and specific investigation of unknown cell‒cell interactions, providing opportunities for further validation compared to traditional approaches.^93^, ^94^, ^95^ Here, we enumerate the cell‒cell communication within DR utilising scRNA‐seq in previous studies, including the enhanced interactions across retinal cells under hyperglycaemic condition as well as crosstalk discovered among cells in FVMs (Figure 4).

In DR, microglia alter ligand expression patterns or secrete angiogenic factors to influence pathogenic angiogenesis and ECs can alter the microglia phenotype in turn.^78^ Ben et al.^50^ deployed a set of ligand–receptor paired interactions in STZ‐induced diabetic and control mice and found that the diabetes‐induced Csf1r‒Csf1 was significantly enriched in microglia and ECs. The following experiments verified that ECs secrete CSF1 which acted on the CSF1R of microglia to activate the MAPK signalling pathway, which elicits microglial differentiation and triggers the secretion of inflammatory factors (Figure 2). The secreted inflammatory factors subsequently foster angiogenesis through a positive feedback mechanism. These results highlight blocking CSF1R signalling to attenuate inflammation‐mediated vascular dysfunction as a possible therapy for DR.

Research on the interactions between microglia and other retinal cells is extensive among the scRNA‐seq studies. Xiao et al.^38^ established single cell libraries using retina from cynomolgus monkeys with spontaneous type 2 diabetes and observed an overall enhancement in communication under hyperglycaemia, with the greatest strength of outgoing and incoming signalling found in microglia. The analysis suggested that an enhanced TNF‐α signalling under hyperglycaemia mediates NF‐κB signalling to consequently activate microglia in an autocrine manner as suggested by Kuno et al.^96^ Communication between microglia and other retinal cells helps suppress excessive activation of microglia. This includes Müller‐derived SPP1 signalling, amacrine‐derived growth arrest specific 6 (GAS6) signalling and cone‐derived transforming growth factor (TGF‐β) signalling, all of which are upregulated under hyperglycaemia. These findings suggest a delicate regulation of microglial activation, paving the way to further exploration of the cellular and molecular mechanisms underlying DR.

To explore the formation of FVMs in PDR, ligand‒receptor analysis was performed on FVM cells. Hu et al.^34^ identified SPP1 as a ligand mainly expressed in activated microglia, contributing to fibroblast‐dominated signalling involved in tissue remodelling and cell‒matrix interactions. Gao et al.^35^ identified three key proteins—CD44, ICAM1 and POSTN—that facilitate interactions among fibroblasts, CD8+ T cells and macrophages and are associated with FVM formation. A study by Xu et al.^36^ revealed APP‐CD74 as the major pathway mediating the interactions of cells on FVMs of PDR in which the signalling was generated mainly from ECs and pericytes and received by most cell populations on FVMs. Corano‐Scheri et al.^37^ conducted a ligand‒receptor analysis between ECs and other cells. ECs communicate with macrophages, pericytes and myofibroblasts via molecules involved in the Notch pathway, which is essential for angiogenesis. In addition, the VEGFC pathway is involved in EC‐myofibroblast crosstalk, revealing a possible previously unrecognised mechanism by which pathological ECs promote the myofibroblast phenotype. Blocking these interactions may prevent the formation of FVMs and avoid severe vision‐threatening complications of proliferative DR.

Taken together, elucidating cell‒cell communication and its alterations in DR contributes greatly to the understanding of disease mechanisms. Further investigations are required to develop novel inhibitory targets for DR treatment.

## 5 scRNA‐seq COMBINED WITH GWAS

GWAS, in which millions of single nucleotide polymorphisms (SNPs) are assayed as genetic variants across many individuals, have helped to lead insights into the architecture of disease susceptibility through the identification of novel disease‐causing genes,^97^, ^98^ and have so far been applied to a variety of diseases including DR.^99^, ^100^, ^101^, ^102^, ^103^

To integrate the studies of GWAS with scRNA‐seq related to DR, researchers have downloaded SNP‒trait associations from the NHGRI‐EBI Catalog of Human GWAS to obtain DR risk‐associated GWAS candidate genes. Module scores were then calculated for gene expression programs in each cell type to identify the cell types most vulnerable to DR.^40^, ^42^ In a study by Chen et al.,^42^ bipolar cells were observed to have the highest association with DR risk followed by amacrine cells. *NRXN3*, a DR risk‐associated candidate gene encoding a neuronal cell surface protein that modulates synapse signals, showed the highest expression in bipolar, amacrine and horizontal cells, but was lower or undetectable in other cell types. These results imply the possible importance of interneurons in the pathogenesis process of DR. Niu et al.^40^ reported rods, cones, rod bipolar cells, vascular ECs and Müller glia as the cell types most closely related to proliferative DR risk.

However, the two current analyses did not consider the risk levels of different variant alleles, and most of the SNPs were localised to noncoding regions of the genome; thus, they could not be interpreted using scRNA‐seq data. Hence, a more scientific and comprehensive combination of the two methodologies is required. Jagadeesh et al.^104^ introduced a framework for integrating scRNA‐seq, epigenomic SNP‐to‐gene maps and GWAS summary statistics to infer the underlying cell types and processes by which genetic variants influence disease, providing a powerful avenue for future research in the field of DR. This combination may point out new directions for investigating DR pathogenesis, especially into the cell types that have been overlooked before.

## 6 FUTURE EXPECTATIONS AND CHALLENGES

The advent of scRNA‐seq has enabled high‐throughput molecular profiling of DR at single‐cellular level and thereby furnished invaluable insights into the complexities of biological systems within general DR. Future research in the field of DR should be directed towards several critical domains.

First, there is an imperative to narrow the scope of inquiry from general DR to the specific subtypes that impact a considerable number of patients yet remain untreatable. Stringent control of blood glucose levels, as measured by haemoglobin A1c (HbA1c) levels below 7%, has been instrumental in diminishing the incidence and progression of general DR, which can drastically reduce the risk of DR development and progression by over 50%.^105^, ^106^ Despite these advancements, a significant proportion of diabetic patients who achieve tight glycaemic control remain at considerable risk for developing DR, a subset often referred to as glucose‐well‐controlled DR.^107^, ^108^ This underscores the critical necessity for future research to pivot towards a precision medicine approach, employing a single‐cell perspective to meticulously investigate the pathogenesis of DR in this subset of patients and finally lead to the development of novel targeted therapeutic strategies.

Subsequently, the utilisation of scRNA‐seq technology should be further developed to incorporate single‐cell and spatial multi‐omics strategies. The scRNA‐seq technology provides information from a transcriptomic perspective while other biological regulating processes before or after transcription are neglected, highlighting the value of utilising other single‐cell omics techniques. Single‐cell epigenomics such as DNA methylation, chromatin accessibility studies, examine the entire genome, including non‐protein coding regions with millions of candidate cis‐regulatory elements, complementing transcriptome analysis by providing insights into cell‐type‐specific gene expression regulation.^109^ Single‐cell proteomics based on mass spectrometry allows for the analysis of more proteins and post‐translational modifications without requiring affinity reagents, helping elucidate the signalling pathways necessary for the function of a single cell.^110^ Boneva et al.^111^ performed the first in‐depth transcriptional and single‐cell proteomic analysis of diabetic retinal neovascularisation tissue samples to characterise the involved cell types, and discovered numerous HLA‐DR+ immune cells co‐expressing α‐SMA suggesting their transdifferentiation to myofibroblasts in retinal neovascularisation. Furthermore, spatial omics has developed rapidly to allow the study of different molecular analytes within their native tissue context, avoiding losing information about physical interactions within and between cells.^112^ Given the retina's distinct layered structure, the integration of scRNA‐seq technology with additional spatial multi‐omics techniques holds the potential to uncover novel mechanisms and therapeutic targets of DR through a multi‐dimensional analytical approach.

Despite the promise of scRNA‐seq in the study of DR, there are several limitations and challenges. One such limitation is the loss of histological context during the procedure of sample preparation. The confounding factors inherent in the processes of digestion, cell isolation and preservation may alter gene expression profile.^12^ This concern was highlighted in a previous study on muscle stem cells, where a specific subpopulation was significantly affected by the widely used dissociation protocol, with analogous clusters detected in other single‐cell datasets, suggesting the influence of procedural artefacts.^113^ Consequently, technical variations, such as laboratories, conditions and sample preparation, may cause discrepancies among the results of different studies.

Another significant challenge in the application of scRNA‐seq to DR research is the difficulty in obtaining human retina tissues for research. Restricted by the acquisition of retinal samples from patients with DR, the majority of single‐cell detection samples currently are derived from the retinas of mice and rats, with only a few reports on human samples using FVMs and PBMCs. There is a notable absence of reports on retinal samples specifically from patients with DR. Animal models provide some fundamental information. However, considering structural differences, such as the absence of macula in the mouse retina, which is a feature present in the human retina, there exist potential biases and necessitate further experimental validation in our understanding of the mechanisms of DR. Besides, the high cost of scRNA‐seq and the difficulty in obtaining human retina tissues impede the large‐scale application in DR. The small sample size limits the generalisability of conclusions and therefore scRNA‐seq cannot be used for studying the disease etiology at a population level so far.

Additionally, the absence of standardised protocols in scRNA‐seq analysis presents significant challenges to the reliability and consistency of integrative analyses conducted across various sequencing platforms. The establishment and development of public databases such as the Gene Expression Omnibus for depositing scRNA‐seq data have remarkably facilitated the sharing of published results. The availability of raw transcriptional files allows for the re‐analysis of existing data, serving as independent cohorts to verify internal findings for new research questions, and integrative methods allow the combination and comparison among different studies to reach higher generalisability of the findings.^114^ However, the heterogeneity in single‐cell solution preparation, experimental conditions and selection of animal species and DR models can introduce nonnegligible discrepancy and incomparability across results, thus the integration of data from diverse sources should be performed with caution.

Finally, despite the wealth of information provided by scRNA‐seq, it only proposes possible assumption for potential mechanisms, necessitating further validation using additional methodologies. More rigorously designed ‘wet’ experiments are required to validate these hypotheses.

## 7 CONCLUSION

In summary, the application of scRNA‐seq has greatly elucidated the potential pathogenic mechanisms underlying DR. This advanced technique empowers researchers to comprehensively investigate DEGs, key cell subpopulations, transition between different cell states and complex cell‒cell communication associated with DR, as well as combine with GWAS to identify cell types most related to DR risk genetic loci. Through integration with other emerging and refined single cell and spatial omics technologies, this approach promises to deepen our understanding of disease mechanisms and hold enormous potential for discovering new therapeutic approaches for DR in the future.

## AUTHOR CONTRIBUTIONS

*Design*: Fang Zhang and Xun Xu. *Writing*: Xinzi Zhang. *Review and editing*: Fang Zhang and Xinzi Zhang. All the authors reviewed and approved the final manuscript.

## CONFLICT OF INTEREST STATEMENT

The authors declare they have no conflicts of interest.

## ETHICS STATEMENT

Not applicable.

## Data Availability

Data sharing not applicable to this article as no datasets were generated or analysed during the current study.
